# Caractéristiques épidémiologiques de l’épidémie de Covid-19 entre 2020 et 2022 au Kongo central, RDC

**DOI:** 10.48327/mtsi.v3i2.2023.356

**Published:** 2023-04-18

**Authors:** El-Mouksitou AKINOCHO, Matthieu KASONGO, Kristel MOERMAN, Felipe SERE, Yves COPPIETERS

**Affiliations:** 1 Université libre de Bruxelles (ULB), École de Santé publique, Centre de recherche Épidémiologie et biostatistique, Route de Lennik 808, 1070 Bruxelles, Belgique; 2 Memisa Belgique ASBL, Square de Meeûs 19, 1050 Bruxelles, Belgique

**Keywords:** Covid-19, Mortalité, Facteurs associés,, Kongo central, République démocratique du Congo, Afrique subsaharienne, Covid-19, Mortality, Associated factors, Kongo central, Democratic Republic of the Congo, Sub-Saharan Africa

## Abstract

**Introduction:**

La République démocratique du Congo (RDC) a connu une transmission communautaire généralisée du SARS-CoV-2 et a enregistré quatre vagues successives de mars 2020 à mars 2022. L'objectif de cette étude est de déterminer les caractéristiques sociodémographiques des patients Covid-19 durant ces vagues épidémiques dans la province du Kongo central et d'identifier les facteurs associés aux décès.

**Matériel et méthodes:**

Il s'agit d'une étude transversale portant sur les données Covid-19 du système de surveillance provincial. Ce sont des données de la surveillance épidémiologique, celles des laboratoires (tests), des données hospitalières et du suivi des malades. Nous avons déterminé les caractéristiques des cas positifs durant toute la période et effectué une régression logistique des facteurs associés aux décès.

**Résultats:**

Au cours des vagues successives, 9 573 cas positifs ont été notifiés dans la province, dont 546 cas lors de la première vague et 6 346 lors de la quatrième vague. Sept cas positifs sur 10 concernaient les personnes âgées de 25 à 64 ans. Les districts de Matadi, Moanda et Mbanza-Ngungu étaient les plus touchés. L’âge supérieur à 64 ans [OR: 7, 2 IC:5, 1-10, 3] et la vague 2 [OR: 4, 4 IC:1, 6-12, 4] sont les facteurs qui sont statistiquement associés aux décès.

**Conclusion:**

Comme dans d'autres contextes africains, ce sont l’âge, les comorbidités, le niveau socio-professionnel plus élevé et le fait d'habiter en milieu urbain, qui ont été les facteurs de risque majeurs de formes graves et de décès. Notre analyse souligne l'importance de recueillir et d'analyser plusieurs variables épidémiologiques jusqu'au niveau provincial au fil du temps pour une meilleure connaissance continue de la situation.

## Abstract (en)

**Introduction:**

The Democratic Republic of Congo (DRC) has experienced widespread community transmission of SARS-CoV-2 and has recorded four successive waves from March 2020 to March 2022. The objective of this study is to determine the socio-demographic characteristics of Covid-19 patients during these epidemic waves in the province of Kongo central and to identify factors associated with deaths.

**Material and methods:**

This is a cross-sectional study of Covid-19 data from the provincial surveillance system. The data consisted of epidemiological surveillance data, laboratory data (tests), hospital data and patient follow-up data. We determined the characteristics of positive cases throughout the period and implemented logistic regression of factors associated with death.

**Results:**

During the successive waves, 9, 573 positive cases were reported in the province, 546 cases in the first wave and 6, 346 in the fourth wave. Seven positive cases out of 10 concerned people aged 25 to 64. The districts of Matadi, Moanda and Mbanza-Ngungu were the most affected. Age above 64 [OR: 7.2 CI:5.1-10.3] and wave 2 [OR: 4.4 CI:1.6-12.4] were the factors statistically associated with death.

**Conclusion:**

As in other African settings, age, comorbidities, higher socio-professional level and living in urban areas were the major risk factors for severe forms of the disease and death. Our analysis underlines the importance of collecting and analysing several epidemiological variables down to the provincial level over time for a better continuous knowledge of the situation.

## Généralités

La crise sanitaire Covid-19 a touché la majorité des régions du monde. En République démocratique du Congo (RDC), la province du Kongo central n'est pas exemptée [7]. Du fait de son impact sur le système de santé ainsi que sur les politiques sociales et économiques, cette pandémie à SARS-CoV-2 a constitué un défi pour le monde entier. Les politiques de santé à travers le monde ont adopté une approche de santé globale avec une certaine harmonisation des mesures. En Afrique, plusieurs initiatives ont été déployées en collaboration avec les partenaires, comme la surveillance active et la mise à disposition des tests de dépistage et des équipements médicaux; la mise en place des mesures barrières afin de garantir la sécurité dans les moyens de subsistance et ne pas bloquer l’économie; et la vaccination [1, 19].

Les prédictions des chercheurs étaient défavorables pour les pays à revenus faibles et intermédiaires du fait du sous-développement des services de santé [2, 21]. Provenant en tout ou en partie d'entités extérieures au continent, ces projections ne tenaient pas suffisamment compte des différences contextuelles entre continents [6, 17, 22]. Le SARS-CoV-2 s'est propagé rapidement en Afrique en peu de temps, mais les taux d'incidence y étaient plus faibles comparés à ceux observés sur les autres continents [20]. Il existe aujourd'hui un manque de connaissances sur les facteurs de cette répartition inégale des cas de Covid-19 dans les pays d'Afrique subsaharienne [12]. Au sein d'un même pays, on a observé une répartition des cas inégale du fait des spécificités de chaque région. Le continent africain se démarque par son hétérogénéité au niveau régional, national et provincial; ces différences sont d'ordre sociodémographique, culturel, climatique, économique et sanitaire [11]. La réponse des pays à la pandémie de Covid-19 a donc été affectée par les caractéristiques de chaque pays [18]. Ces différences justifient l'importance des recherches qui s'appuient sur les données brutes au lieu de données agrégées qui peuvent déformer la compréhension épidémiologique dans chaque contexte.

Le Ministre de la Santé publique, hygiène et prévention (MSPHP) de la RDC a officiellement déclaré l’épidémie le 10 mars 2020 [8, 16]. La RDC a connu une transmission communautaire du SARS-CoV-2 et a déjà enregistré quatre vagues successives depuis son émergence jusqu’à ce jour (mars 2022). Elles sont survenues dans un contexte de crise humanitaire prolongée, exacerbée par la récurrence des conflits armés à l'est du pays, provoquant d'importants déplacements de populations et un faible accès aux services de base. En RDC, la stratégie de lutte contre la Covid-19 a été élaborée à partir des recommandations internationales et des succès d'autres pays. Après la mise en œuvre, certaines orientations stratégiques ont été modifiées et améliorées. Souvent, les stratégies mises en place ont été basées sur peu de données probantes. Il y a donc un véritable besoin de recherche et d'accroissement des connaissances empiriques sur ces événements de santé publique.

La province du Kongo central (anciennement appelé Bas-Congo) a été créée en 1962. Elle est très développée économiquement, avec un accès à la mer qui favorise l'importation et l'exportation des marchandises. La population totale est composée de 221 223 habitants en 2022. La proportion de la population accessible au 1^er^ échelon est de 93.2% et celle accessible au 2^e^ échelon de 80.3% (source: extrapolation zonale). 35% de la population n'a pas accès à l'eau potable ou à une source d'eau aménagée et 20% de la population est située dans un rayon de plus de 40 km par rapport à l'hôpital de référence. La province comptait 17 aires de santé en 2019, contre 18 en 2022. Les ressources sanitaires au début de la crise Covid-19 étaient basées sur une première ligne de soins de santé primaire assez performante, avec des taux d'utilisation des services supérieurs à 80% (consultations curatives, prénatales…) [4]. Les partenaires de la province sont intervenus dans la riposte contre la Covid par différentes interventions: renforcement des compétences des prestataires, hygiène, intrants de protection, installation de citernes aériennes. Au niveau communautaire, il y a eu redynamisation du comité de riposte dans toute la province. Les répercussions de la crise sur le système de santé ont été entre autres: diminution des accouchements assistés, diminution des consultations prénatales, et mise à mal de la continuité thérapeutique avec des soucis de prise en charge (exemple du manque d'héparine dans la prévention des complications vasculaires et de son prix excessif pour les malades). Le plateau technique des hôpitaux a été insuffisant avec un manque d'extracteurs d'O_2_ qui sont seulement arrivés au début de la 3^e^ vague. De plus il y a eu en démarrage de crise de grosses erreurs dans la prévention des contaminations par exemple au laboratoire de l'hôpital, les mauvaises conditions d'isolement des malades, etc. La vaccination contre la Covid-19 (vaccin Pfizer) a commencé dans la province à partir de mai 2021, mais l'adhésion de la population a réellement démarré en juillet 2021: 400 personnes étaient vaccinées à cette date. Avec l'arrivée du vaccin de Johnson, il y a eu une meilleure adhésion: 2215 personnes vaccinées en septembre 2021 [1].

L'objectif de cette étude était de déterminer les caractéristiques sociodémographiques des patients Covid-19 durant les 4 vagues épidémiques dans la province du Kongo central et d'identifier les facteurs associés aux décès afin d'améliorer l’état de santé et le bien-être des populations selon leurs spécificités et de fournir des données qui permettront d'anticiper les pandémies futures et de renforcer le système de santé local.

## Méthodes

### Type d’étude et sources de données

Il s'agit d'une étude transversale portant sur les données Covid-19 (surveillance épidémiologique, celles des laboratoires (tests) et du suivi des malades). Les données des laboratoires donnent accès aux tests de dépistage réalisés, les types de test et leurs résultats. Le suivi des malades permet d'analyser l'issue des cas positifs et les données épidémiologiques de surveillance donnent accès à des données agrégées sur les cas et leurs caractéristiques sociodémographiques. La documentation grise et des données communautaires ont permis de tracer une « ligne du temps » de la crise Covid-19 reprenant les grands événements et les étapes clés concernant la province.

Toutes les personnes ayant contracté le virus SARS-CoV-2 durant les quatre premières vagues, détectées dans la Province, ont été incluses dans cette étude, sans distinction d’âge, de sexe, de catégorie socioprofessionnelle et de lieu résidence.

### Validité des données

Les 3 sources d'information proviennent de systèmes de récolte de données standardisés et existant avant la crise. Les données de laboratoire proviennent de deux laboratoires disposant de la méthode RT-PCR (Reverse transcription-polymerase chain reaction) dont les données sont transmises au niveau central dans le cadre de la surveillance épidémiologique. Les tests sont réalisés suivant des protocoles nationaux. Le diagnostic de confirmation de la Covid-19 se fait donc principalement par la méthode RT-PCR avec ses différentes variantes (manuelle, GeneXpert, Abbott, ABI/Biorad), mais aussi à travers les TDR-Ag (Panbio/Abbott, Standard Q/Biosensor/Ichroma II). Les tests sont gratuits et les prélèvements se font dans les structures de soins (centre de santé de référence ou hôpital).

Les données hospitalières viennent des bases de données des différents hôpitaux, auxquelles des indicateurs spécifiques à la Covid-19 ont été ajoutés en début de pandémie. Ces données hospitalières sont transmises de façon hebdomadaire à la province, qui les analyse avec les données de surveillance épidémiologique de la première ligne de soins. Ce sont des données exhaustives et qui bénéficient d'un protocole de récolte de données et d'assurance qualité bien antérieur à la crise.

### Variables d’étude

Trois types de données ont été analysées dans ces bases: (i) les données sociodémographiques comme l’âge (en années), le sexe et la zone sanitaire; (ii) les données des laboratoires comprenant le nombre de tests réalisés, le type de test (PCR, test antigénique) et le nombre de cas positifs; (iii) les données sur l’évolution de la maladie concernent l'issue du cas (décès ou vivant).

### Analyse des données

Nous avons réalisé l'analyse descriptive des variables quantitatives avec la moyenne, l’écart-type comme indicateurs de tendance centrale (avec représentation graphique par des boîtes à moustache); les variables catégorielles avec les fréquences absolues et relatives. Des cartographies ont été réalisées pour étudier la distribution spatio-temporelle des cas dans la province. A ensuite été effectuée une régression logistique univariée de la variable dépendante qualitative (décès) sur chaque variable indépendante (l’âge, le sexe et la vague). Les odds ratios ont été rapportés pour déterminer la force d'association des facteurs de risque. Pour identifier les facteurs de confusion et faire une analyse prédicitive, un modèle de régression logistique a été élaboré. Le test d'Hosmer et Lemeshow a été utilisé pour étudier l'adéquation des modèles aux données. Tous les tests ont été effectués en bilatéral au seuil alpha de 5%.

## Résultats

### Caractéristiques de la population au cours des 4 vagues

**Figure 1:**
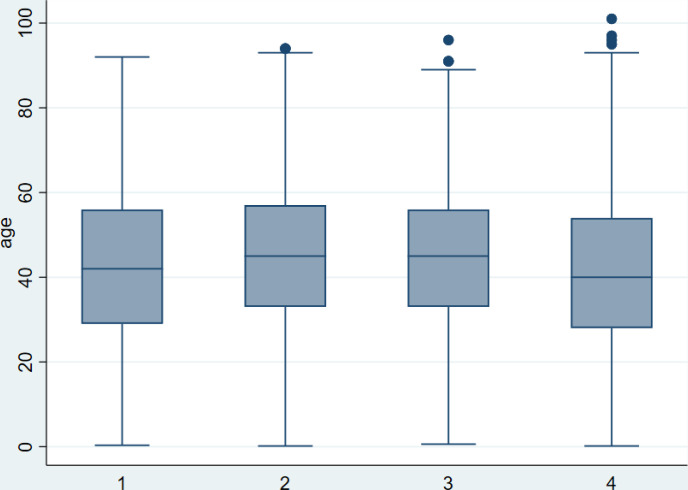
Distribution de l’âge des cas positifs Covid-19 par vague dans la province du Kongo central (mars 2020 à mars 2022) Age distribution of Covid-19 cases by wave in Kongo central province (March 2020 to March 2022)

Durant les deux années de suivi (mars 2020 à mars 2022), 9 573 cas positifs ont été notifiés au Kongo central, dont 546 cas lors de la première vague, et 6 346 lors de la quatrième vague (la complétude des données est considérée comme bonne). La distribution de l’âge était similaire au cours des quatre vagues, 7 cas positifs sur 10 concernaient les personnes âgées de 25 à 64 ans, la médiane de l’âge variait autour de 40 ans durant les 4 vagues, 50% des cas positifs avaient entre 30 et 55 ans (Fig. 1).

Les quatre vagues analysées sont attribuées à des variants différents (majoritairement): Alpha (B.1.1.7) pour la 1^re^, Bêta/Gamma (P1) pour la seconde, Delta (B1.617.2) pour la 3^e^ et Omicron (BA.2, BA.4-5) pour la dernière. Au cours de la 4^e^ vague, la proportion des jeunes (15-24 ans) atteints a doublé comparée à la 3^e^ vague (7,4% *vs* 14, 1%). On note une légère prédominance masculine au cours des vagues, à l'exception de la vague 4 où les proportions des hommes et des femmes sont similaires (48, 6% *vs* 48,9%). Dans la province du Kongo central, la zone sanitaire Matadi est la plus touchée (zone portuaire, capitale de la province). Quant à la deuxième zone sanitaire la plus touchée, cela dépend des vagues: Nzanza pour la 1^re^ vague, Moanda pour les 2^e^ et 3^e^ vagues, et Boma pour la 4^e^ vague. La PCR est le test le plus réalisé lors de la première vague, les tests antigéniques (TDR) sont les plus utilisés lors des autres vagues. Au cours des 4 vagues, la proportion de décès est faible, on note 135 (1, 2%) décès enregistrés, la vague 2 ayant été la plus meurtrière avec une proportion de 3, 4% (Tableau I).

**Tableau I:** Caractéristiques des patients Covid-19 par vague dans la province du Kongo central (mars 2020 à mars 2022) Characteristics of Covid-19 patients by wave in the province of Kongo central (March 2020 to March 2022)

Variables	Vague 1 n = 546 (%)	Vague 2 n = 1 242 (%)	Vague 3 n = 1 390 (%)	Vague 4 n = 6 346 (%)	Total n = 9 573 (%)
**Âge en années (moyenne ± écart-type)**	42, 2±17,8	45, 1±17,3	44, 9±16,5	41, 3±17,7	42, 4±17,5
0-4 ans	6 (1, 1)	15 (1, 2)	7 (0, 5)	36 (0, 6)	65 (0, 7)
5-14 ans	26 (4, 8)	26 (2, 1)	24 (1, 7)	224 (3, 5)	309 (3, 2)
15-24 ans	55 (10, 1)	114 (9, 2)	103 (7, 4)	894 (14, 1)	1 176 (12, 3)
25-64 ans	386 (70, 7)	929 (74, 8)	1078 (77, 6)	4350 (68, 6)	6 767 (70, 7)
65+ ans	54 (9, 9)	18 (12, 7)	177 (12, 7)	682 (10, 8)	1 076 (11, 2)
manquantes	19(3, 5)	-	1(0, 1)	160 (2, 5)	180 (1, 9)
**Sexe**					
masculin	339 (62, 1)	712 (57, 3)	805 (57, 9)	3 085 (48, 6)	4 967 (51, 9)
féminin	196 (35, 9)	529 (42, 6)	584 (42, 0)	3 104 (48, 9)	4 436 (46, 3)
manquantes	11 (2, 0)	1 (0, 1)	1 (0, 1)	157 (2, 5)	170 (1, 8)
**District**					
Matadi	219 (40, 2)	365 (29, 4)	420 (30, 3)	1 120 (17, 8)	2 129 (22, 4)
Moanda	39 (7, 2)	198 (16)	331(23, 9)	555 (8, 9)	1 124 (11, 8)
Boma	16 (3)	74 (6)	59 (4, 3)	802 (12, 8)	954 (10, 1)
Kwilu-Ngongo	9 (1, 7)	43 (3, 5)	112 (8, 1)	603 (9, 6)	770 (8, 1)
Mbanza-Ngungu	33 (6, 1)	159 (12, 9)	74 (5, 4)	425 (6, 8)	693 (7, 3)
Nzanza	105 (19, 3)	58 (4, 7)	118 (8, 5)	276 (4, 4)	557 (5, 9)
Kimpese	43 (7, 9)	66 (5, 4)	33 (2, 4)	233 (3, 7)	378 (4)
Kisantu	9 (1, 7)	29 (2, 4)	72 (5, 2)	191 (3, 1)	301 (3, 2)
Lukula	12 (2, 2)	61 (5)	13 (1)	261 (4, 2)	348 (3, 7)
Luozi	1 (0, 2)	25 (2, 1)	2 (0, 2)	221 (3, 6)	249 (2, 7)
Nsona-Mpangu	18 (3, 3)	22 (1, 8)	26 (1, 9)	134 (2, 2)	205 (2, 2)
Seke-Banza	2 (0, 4)	6 (0, 5)	2 (0, 2)	217 (3, 5)	227 (2, 4)
autres ZS	40 (7, 4)	136 (11)	128 (9, 3)	1 268 (20, 2)	1 598 (16, 8)
**Test**					
TDR	156 (28, 6)	864 (69, 6)	1 383 (99, 5)	5 785 (91, 2)	8 221 (85, 9)
PCR	369 (67, 6)	330 (26, 6)	3 (0, 2)	558 (8, 8)	1 276 (13, 3)
TDR+PCR	9 (1, 7)	5 (0, 4)	-	-	14 (0, 1)
manquantes	12 (2, 2)	43 (3, 4)	4 (0, 3)	3 (0, 0)	62 (0, 7)
**Décès**					
oui	4 (0, 7)	43 (3, 4)	9 (0, 7)	79 (1, 2)	135 (1, 4)
non	535 (98, 0)	1198 (96, 5)	1379 (99, 2)	6265 (98, 7)	9426 (98, 5)
manquantes	7 (1, 3)	1 (0, 1)	2 (0, 1)	2 (0, 0)	12 (0, 1)

ZS: zone sanitaire

### Évolution des cas positifs au cours des 4 vagues

**Figure 2:**
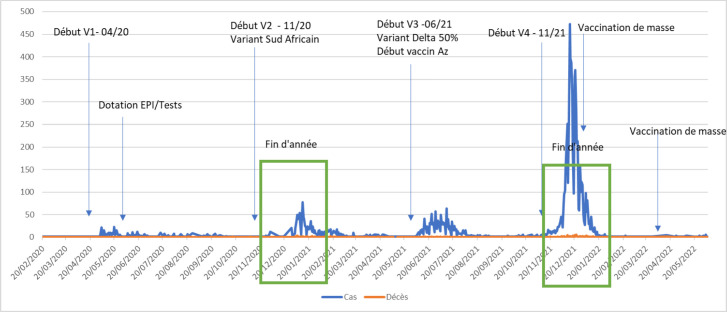
Évolution du nombre de cas positifs (bleu) et du nombre de décès (rouge) Covid-19 durant les quatre vagues dans la province du Kongo central (mars 2020 à mars 2022) Evolution of the number of Covid-19 cases (blue) and deaths (red) during the four waves in Kongo central province (March 2020 to March 2022)

La première vague a débuté en avril 2020, les autres vagues ont commencé environ 6 mois après la vague précédente. Les périodes de fin d'année marquaient aussi les vagues 2 et 4, cette dernière étant la plus importante avec un pic de 470 cas positifs en 24 heures (Fig. 2).

**Figure 3:**
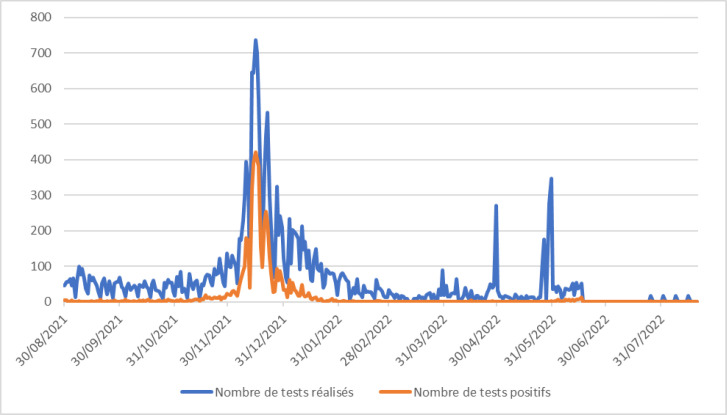
Évolution du nombre de tests Covid réalisés (bleu) et du nombre de cas positifs (rouge) au cours de la quatrième vague dans la province du Kongo central) Evolution of the number of Covid tests performed (blue) and of the number of positive cases (red) during the fourth wave in the province of Kongo central

**Figure 4:**
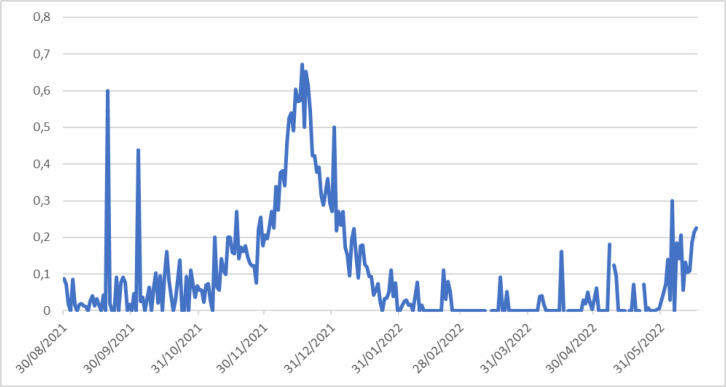
Évolution du taux de positivité Covid-19 au cours de la quatrième vague dans la province du Kongo central Evolution of the Covid-19 positivity rate during the fourth wave in the province of Kongo central

Le nombre de tests réalisés et le taux de positivité sont plus élevés durant la période de fin d'année (Fig. 3). Quelle que soit la vague, le district de Matadi était le plus touché par la Covid-19. Au cours des 2^e^ et 3^e^ vagues, les cas positifs ont été plus élevés dans les districts de Mbanza-Ngungu et Moanda. La 4^e^ vague a touché presque tous les districts, mais les districts de Mbanza-Ngungu, Boma et Matadi étaient les plus touchés (Fig. 4).

La Covid-19 est perçue comme une invention humaine, une « invention de l'homme blanc » fabriquée dans un laboratoire européen. Dans la même logique, elle est également considérée comme une arme biologique contre certains États du monde, notamment ceux en conflit politique et économique. Parler d'une invention humaine pour les épidémies n'est pas une première dans l'histoire des épidémies en RDC. Lors de la récente épidémie d’Ébola dans l'est du pays, c'est une des formes de désinformation qui a conduit les communautés à résister fortement aux actions de réponse humanitaire. Une situation similaire a été observée lors des activités de vaccination, où à l’époque, certains ménages ont résisté à la vaccination de leurs enfants. D'une part, les populations sont insuffisamment informées ou sensibilisées, d'autre part, les vaccins sont considérés comme expérimentaux et ayant des effets négatifs (exemple des vaccins contre Ébola). Tout ceci conduit la population à se fier beaucoup plus aux différentes désinformations qui circulent dans l'environnement qu'aux sources fiables.

### Facteurs associés aux décès

L’âge et la vague sont les facteurs qui sont statistiquement associés aux décès. Les personnes âgées ont 7 fois plus de risque de décéder comparées aux personnes âgées de 25 à 64 ans, après ajustement sur le sexe et la vague. Le risque de décès ajusté sur l’âge et le sexe est 4 fois plus élevé lors de la vague 2, la plus meurtrière des quatre, comparée à la vague 1 (Tableau II).

**Tableau II:** Analyse univariée et multivariée des facteurs associés aux décès Covid-19 dans la province du Kongo central (mars 2020 à mars 2022) Univariate and multivariate analysis of factors associated with Covid-19 death in the province of Kongo central (March 2020 to March 2022)

Variables	OR brut [IC 95%]	p	OR ajusté* [IC 95%]	p
**Âge**				
**5-14 ans**	1, 05 [0, 33-3, 39]	0,923	1, 14 [0, 36-3, 67]	0,823
**15-24 ans**	0, 09 [0, 01-0, 66]	0,018	0, 10 [0, 01-0, 70]	0,021
**25-64 ans**	Réf.		Réf.	
**65+ ans**	7, 29 [5, 14-10, 35]	<0,001	7, 21 [5, 07-10, 27]	< 0,001
**Sexe**				
**féminin**	Réf.		Réf.	
**masculin**	1.47 [1, 04-2, 10]	0,028	1, 21 [0, 85-1, 74]	0,288
**Vague**				
**1**	Réf.		Réf.	
**2**	4.80 [1, 71-13, 44]	0,003	4, 40 [1, 55-12, 41]	0,005
**3**	0.87 [0, 27-2, 84]	0,822	0, 75 [0, 22-2, 48]	0,642
**4**	1.68 [0, 61-4, 62]	0,310	1, 68 [0, 61-4, 65]	0,315

* n = 9195

p < 0, 001 R = 12, 64%

## Discussion

Cette étude rapporte les caractéristiques des cas positifs et l’évolution de la pandémie au cours des 4 vagues dans la province du Kongo central en RDC. Depuis avril 2020, la pandémie a trouvé un terrain fragilisé dans cette province, fragilisé par les maladies infectieuses tropicales (paludisme) et une sous-utilisation des services de santé. Le 1^er^ cas de Covid-19 a été identifié dans la province le 12 mai 2020. L’épidémie a concerné toute la province mais ce sont les zones de Mbanza-Ngungu et de Matadi (épicentre de l’épidémie de la province) qui ont été les plus touchées avec une croissance exponentielle au fil des vagues. On note aussi une transmission communautaire active et soutenue du virus dans les districts avec une forte densité de la population. Le profil épidémiologique des personnes atteintes est caractérisé par les hommes ou les femmes âgées de 25 à 64 ans résidant dans une zone urbaine à plus forte densité. Ce profil est aussi rapporté dans plusieurs autres études [5, 9, 14]. Il semble que les cas de Covid-19 étaient davantage localisés dans les zones résidentielles que dans les zones moins peuplées. Dans la province, même si la 4^e^ vague a été plus importante en termes de contaminations (variant Delta/Omicron), c'est au cours de la 2^e^ vague (variant sud-africain) que nous observons le plus haut taux de létalité, surtout chez les personnes âgées.

Face à l'inconnu, il y a eu des perceptions communes de la population sur la Covid-19: entre le déni de l'existence de la maladie, les influences socio-financières, l'importance des mesures barrières et le comportement à risque de la population… il a fallu un certain temps pour faire comprendre la problématique et favoriser les mesures de protection. Cependant, les soins préventifs en général ont été peu affectés par la crise et tous les indicateurs sont restés stables excepté l'utilisation de la consultation curative et l'occupation des lits hospitaliers (en 2021). D'autres facteurs comme l'augmentation des prix des consultations en général, ont aussi influé sur la diminution de l'utilisation de certains services.

Les différences observées entre les pays d'Afrique et ceux d'autres continents peuvent s'expliquer par l'importation tardive de cas dans le continent, ce qui a favorisé une mise en œuvre précoce des stratégies de surveillance et de prévention. Par rapport à d'autres continents, cela a fait gagner du temps à certains pays pour renforcer leur capacité de détection et de réponse. Cette pandémie a néanmoins plusieurs répercussions sur le système de santé en RDC. La peur persistante de l'infection a entraîné une sous-utilisation des services de santé, en particulier les services de maternité, une perte de chance pour les patients, notamment pour les traitements de longue durée et les patients qui nécessitent un plateau technique avancé pour leur prise en charge.

Le taux de mortalité brute de la province du Kongo central est bas comparé au reste des pays d'Afrique et au reste du monde [2]. Cela pourrait s'expliquer par la population qui est jeune dans cette région et une plus faible prévalence des comorbidités. L’âge avancé augmente le risque de décès, en raison du système immunitaire plus faible qui rend ces personnes plus vulnérables aux infections et aux complications [13]. Le variant Bêta qui circulait pendant la vague la plus importante a été reconnu comme virulent comparé aux autres [10]. Mwenda *et al.* ont rapporté que cette souche a des capacités pour réduire l'immunité naturelle et l'efficacité du vaccin [15]. Une autre étude transversale à l’échelle de l'Afrique subsaharienne a montré que les hommes avaient plus de risque de complications et de décès comparés aux femmes. Selon les auteurs, ce risque accru était probablement dû aux différences entre les facteurs biologiques, sociaux et comportements qui y sont associés [3].

La stratégie de lutte contre la Covid-19 en RDC ayant été élaborée en moins de deux mois, il est pratiquement impossible de rassembler toutes les données existantes à ce sujet. Les approches basées sur les données probantes requièrent néanmoins de meilleures données disponibles.

### Limites de l’étude

Nous avons utilisé l'ensemble des données accessibles au niveau départemental pour la province du Kongo central, mais nous ne pouvons pas nous assurer de la qualité des données et de leur degré d'exactitude. Concernant la représentativité des données, elle est partielle et meilleure pour les données des vagues 3 et 4 que pour la première période de la crise. En effet, on peut facilement considérer une sous-estimation des données (cas, morbidité et décès) pour différentes raisons: le faible accès aux tests diagnostiques lors des 2 premières vagues, la crainte de stigmatisation et d'isolement à l'hôpital, les fausses croyances sur l'origine et les conséquences de la maladie. Des données sur les comorbidités, le niveau socio-économique et d'autres variables n’étaient pas disponibles pour permettre une analyse plus détaillée. Concernant les décès, les critères permettant d'attribuer à la Covid la cause de la mort n'ont pas été standardisés en début de crise. L'analyse des facteurs associés aux décès pourrait être biaisée par les patients qui ont développé des formes graves sans se rendre à l'hôpital. Malgré les limites liées à la qualité des données analysées, les résultats de cette étude pourront servir de comparaison avec les prédictions initiales afin de les améliorer et aider les politiques à anticiper des pandémies surtout dans les zones plus à risque.

## Conclusion

La province du Kongo central n'a pas été épargnée par la pandémie de Covid-19, les districts de Matadi, Mbanza-Ngungu et Moanda ayant été les plus touchés. Les personnes âgées et la « vague 2 » étaient les deux facteurs qui étaient associés aux décès. Sur la base des résultats, il est possible de proposer quelques pistes de renforcement du système à différents niveaux: renforcer la surveillance (notification des cas et décès) communautaire et l'implication de la population dans les choix stratégiques, améliorer les conditions de travail et mieux protéger le personnel de santé et mieux le motiver, accroître les mesures d'hygiène hospitalière et travailler sur une meilleure coordination de l'acheminement des ressources. Cette étude pourra en outre éclairer les politiques sur les interventions mises en place pendant cette pandémie, leur permettant de savoir lesquelles doivent être maintenues ou améliorées. Ainsi les résultats pourront-ils les aider à anticiper d'autres pandémies et à agir dès le début sur les zones à forte transmission.

## Contribution Des Auteurs

El-Mouksitou AKINOCHO a réalisé les analyses et contribué à la rédaction, Matthieu KASONGO a effectué les récoltes de données sur le terrain et a participé aux analyses, Kristel MOERMAN a conçu la recherche, participé à l'analyse et l’écriture, Felipe SERE a conçu la recherche, participé à l'analyse et l’écriture, Yves COPPIETERS a été l'initiateur de la recherche, a effectué les récoltes de données, les analyses et l’écriture de l'article.

## Liens D'intérêts

Les auteurs ne déclarent aucun lien d'intérêt.
